# Robot-assisted and laparoscopic vs open radical prostatectomy in clinically localized prostate cancer: perioperative, functional, and oncological outcomes: A Systematic review and meta-analysis

**DOI:** 10.1097/MD.0000000000015770

**Published:** 2019-05-31

**Authors:** Lan Cao, Zhenyu Yang, Lin Qi, Minfeng Chen

**Affiliations:** a Xiangya School of Medicine; b Department of Urology, Xiangya Hospital, Central South University, Changsha, Hunan, China.

**Keywords:** prostate cancer, radical prostatectomy, robotics, laparoscopy, meta-analysis

## Abstract

**Background:**

To perform a systematic review and meta-analysis evaluating the perioperative, functional, and oncological outcomes and cost of robot-assisted radical prostatectomy (RARP), or laparoscopic radical prostatectomy (LRP) comparing with open radical prostatectomy (ORP) in men with clinically localized prostate cancer through all prospective comparative studies.

**Methods:**

A comprehensive literature search was performed in August 2018 using the Pubmed, Medline, Embase, and Cochrane databases. Only randomized controlled trials (RCTs) and prospective studies including patients with clinically localized prostate cancer were eligible for study inclusion. Cumulative analysis was conducted using Review Manager v. 5.3 software.

**Results:**

Two RCTs and 9 prospective studies were included in this systematic review. There were no significant differences between RARP/LRP and ORP in overall complication rate, major complication rate, overall positive surgical margin (PSM) rate, ≤pT2 tumor PSM rate, ≥pT3 tumor PSM rate. Moreover, RARP/LRP and ORP showed similarity in biochemical recurrence (BCR) rate at 3, 12, 24 months postoperatively. Urinary continence and erectile function at 12 months postoperatively between RARP and ORP are also comparable. RARP/LRP were associated with significantly lower estimated blood loss [mean difference (MD) −749.67, 95% CI −1038.52 to −460.82, *P* = .001], lower transfusion rate (OR 0.17, 95% CI 0.10 to 0.30, *P* < .001) and less hospitalization duration (MD −1.18, 95% CI −2.18 to −0.19, *P* = .02). And RARP/LRP required more operative time (MD 50.02, 95% CI 6.50 to 93.55, *P* = .02) and cost.

**Conclusion:**

RARP/LRP is associated with lower blood loss, transfusion rate and less hospitalization duration. The available data were insufficient to prove the superiority of any surgical approach in terms of postoperative complications, functional and oncologic outcomes.

## 1 Introduction

Prostate cancer (PCa) is the second most frequently diagnosed cancer and the fifth leading cause of cancer death affecting men worldwide, in 2018, estimated new PCa cancer cases was 1,276,106 worldwide and 358,989 of total PCa cancer deaths in men.^[1]^ Most countries experienced PCa increases in incidence in the recent years.^[2]^ Currently, men diagnosed with clinically localized prostate cancer have a variety of management options including radical prostatectomy (RP), RP is recommended for localized prostate cancer patients with a life expectancy >10 years as a first-line treatment.^[3,4]^

Open radical prostatectomy (ORP) is the standard procedure for the treatment of clinically localized prostate cancer; however, this procedure is associated with considerable blood loss, postoperative pain and long hospitalization duration. Laparoscopic radical prostatectomy (LRP) was first reported in the early 1990s, with the aim of reducing postoperative pain, postoperative morbidity and allowing faster recovery.^[5,6]^ Therefore, LRP has become an alternative standard procedure for RP. However, some limitations of LRP resulted in a long-term learning curve for urologists,^[7]^ which hindered LRP application worldwide. Alternatively, the robotic-assisted radical prostatectomy (RARP) was introduced in the 2000s, which could reduce the technique challenge of laparoscopic procedures, therefore shortened the learning time.^[8]^ RARP o-ers some advantages compared with standard laparoscopy, including articulated instruments, tremor filtration and three-dimensional visualization. Therefore, RARP has been widely adopted as a standard procedure for clinically localized PCa worldwide.^[9]^

Several systematic reviews have compared RARP/LRP and ORP; however, the results have been inconsistent. Some studies reported RARP/LRP were related to less blood loss, lower transfusion rate, shorter hospitalization duration, better functional and oncological outcomes, while others have failed to find these relationships; Importantly, none of them compared long-term oncological outcomes.^[10–20]^ Most of these systematic reviews included lots of retrospective studies with low quality.^[11–20]^ Du et al^[11]^ recently reported that RARP could provide better functional outcomes including urinary continence and potency, however, they included a lot of retrospective studies with low quality; also, they did not provide biochemical recurrence (BCR) rate following RARP, which is an important long-term oncological outcome assessing safety of RARP. Ilic et al^[10]^ included only randomized controlled trials (RCT), while the RCT quantities were hardly enough to get any conclusions. Therefore, this systematic review and meta-analysis including prospective comparative studies was conducted, comparing RARP/LRP to ORP in perioperative, functional and oncologic outcomes and cost, especially long-term follow-up BCR, to provide valuable insights into the appropriate choice of surgical procedures for clinically localized PCa patients.

## 2 Materials and methods

### 2.1 Literature search strategy

We performed literature search in PubMed, Medline, EMBASE, and Cochrane Central Register of Controlled Trials databases. Studies comparing RARP or LRP with ORP were searched (see supplementary): prostate cancer, open radical retropubic prostatectomy, laparoscopic radical prostatectomy, robot-assisted radical prostatectomy, robot-assisted laparoscopic prostatectomy, minimally invasive radical prostatectomy, randomized controlled trial, prospective controlled trial. No date, language and publication status limits were applied. Additionally, relevant reviews and references of all included articles were searched manually.

### 2.2 Criteria for considering studies for this review

Randomized controlled trials (RCTs) and prospective non-randomized comparative studies comparing RARP or LRP with ORP in perioperative, oncologic, and functional outcomes and cost, reporting at least one outcome of interest, patients diagnosed with clinically localized PCa were selected. Patients with PSA level > 20 ng/ml before surgery were excluded.

### 2.3 Study screening

Two authors (LC and ZY) independently reviewed all records from the search. Discrepancy was resolved through open discussion. Studies in non-English languages or published as reports and meeting abstracts were excluded in this systematic review. Articles were screened with titles and abstracts for further review, and then potential articles were reviewed. All studies included in this systematic review were reviewed and evaluated based on PRISMA (See supplementary).^[21]^

### 2.4 Data extraction

A data extraction form was designed, and data extraction was performed by 2 investigators (LC and ZY) independently. A domain-based risk of bias evaluation was conducted on RCTs as described in the Cochrance Handbook for Systematic Reviews of Interventions.^[22]^ Disagreements were solved by MC. This tool includes assessment of sequence generation, blinding of participants, allocation concealment, incomplete outcome data, personal and outcome, selective outcome reporting and other sources of bias. The quality of the evidence was assessed using Grading of Recommendations Assessment, Development and Evaluation (GRADE).^[23]^ Risk of bias and quality of evidence were individually assessed by LC and ZY, disagreements were resolved by MC.

We extracted the study characteristics and participants’ characteristics from the included studies. Perioperative characteristics including blood loss, operative time, transfusion rate, hospitalization duration, catheterization duration, overall complications and major complications; Functional outcomes including erectile dysfunction and urinary continence; Oncologic outcomes including positive surgical margin (PSM) and biochemical recurrence (BCR); Patient total cost was also extracted.

### 2.5 Statistical analysis

For purpose of analysis, RARP/LRP was considered the experimental intervention, and ORP was considered the control intervention. Continuous outcomes and dichotomous variables were reported as mean differences (MD) and odds ratios (OR) respectively, with 95% confidence interval (CI). Heterogeneity was assessed using the I^2^ statistic and *P* value.^[24,25]^ A random-effects model was applied if *P* < .1 or I^2^ > 25%, otherwise, a fixed-effects model was applied. The Mantel-Haenszel (M-H) method was used for meta-analysis of dichotomous variables, and the inverse variance method was used for calculation of continuous outcomes. Publication bias was assessed by assessing visual symmetry of funnel plots if there are enough studies.

Review Manager V5.3 software (Cochrane Collaboration, Oxford, UK) was used for cumulative analysis. Considering the limitations of the Cochrane software,^[19]^ only data which was reported as means and standard deviations (SDs), or data could be transferred to means and SDs were pooled for continuous variables. Statistically significant was considered with a 2-sided *P* < .05.

## 3 Results

**Figure 1:**
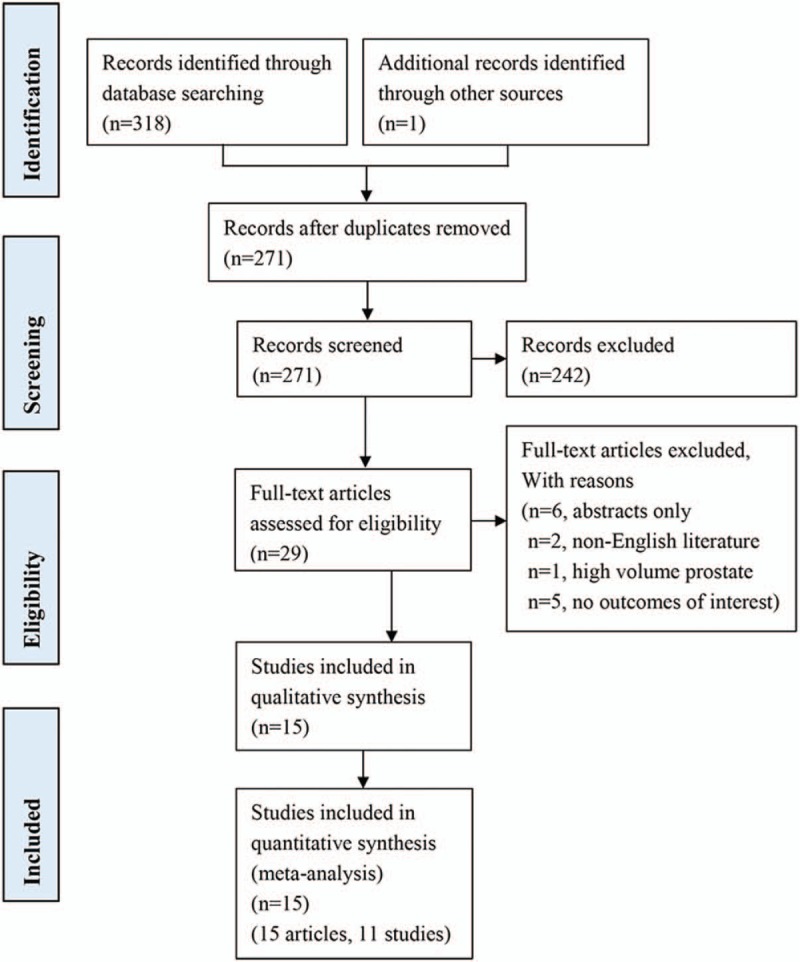
Flow diagram.

The literature search retrieved 271 unduplicated records. Two hundred and forty-two articles were excluded after titles or abstracts screening, 29 articles remained for further review. Subsequently, 14 articles were excluded after full text review. Eventually, 15 articles that met eligibility criteria were included in the systematic review and meta-analysis.^[26–40]^ The process of screening articles for selective records is illustrated in Figure 1. Of these included studies, 2 articles^[28,29]^ were based on the same RCT and reported short-term and long-term outcomes, respectively, and a prospective non-randomized LAPPRO study were reported on 4 different articles.^[26,27,31,32]^

### 3.1 Characteristics of included studies

**Figure 2:**
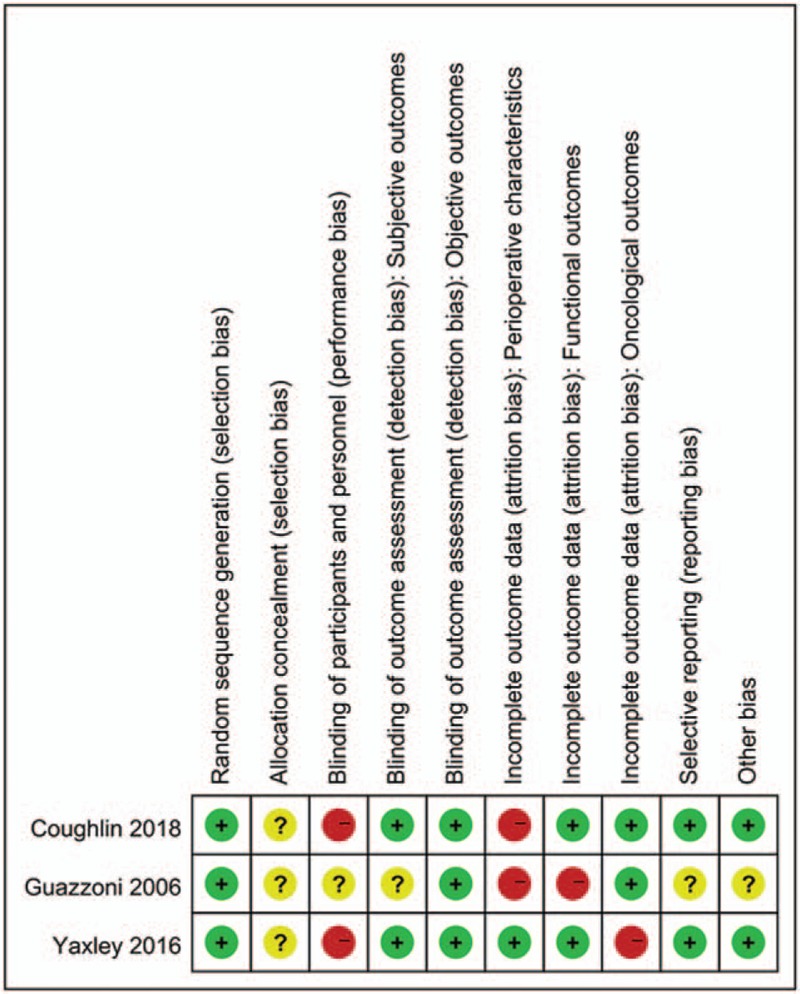
Risk of bias for RCTs.

The 2 authors who extracted data were in total agreement. Characteristics of included studies are summarized in Table 1. Most of the studies are prospective non-randomized studies,^[26,27,30–37,39,40]^ including only 2 RCTs (3 articles).^[28,29,38]^ Six studies (9 articles) compared outcomes between RARP and ORP^[26–29,31,32,34,35,39]^; 5 studies compared LRP and ORP.^[33,36–38,40]^ Among the totally 8522 patients included in this study, 5051 patients underwent RARP or LRP, and 3471 underwent ORP. Overall, both RCTs were assessed as to be at moderate risk of bias (Fig. 2).

**Table 1:**
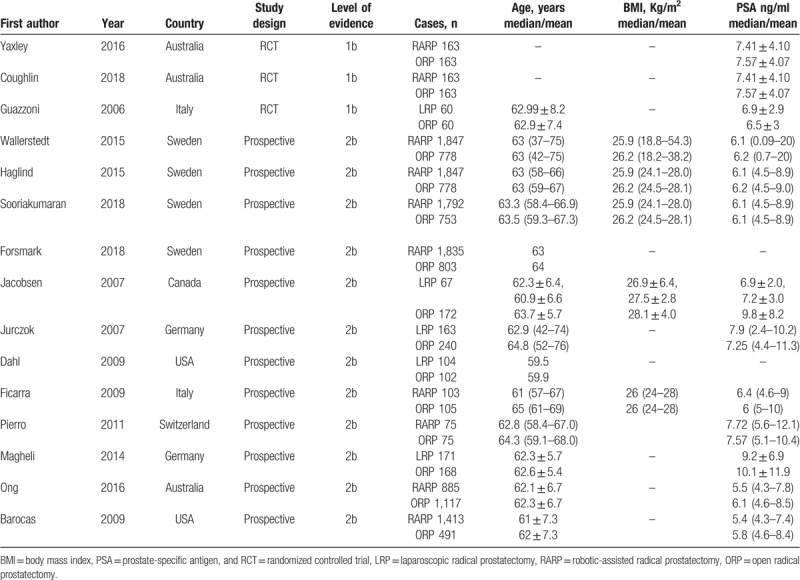
Characteristics of studies included in the systematic review and meta-analysis.

### 3.2 Perioperative outcomes and complications

Perioperative characteristics comparing RARP/LRP with ORP, including operative time, blood loss, transfusion rate, hospitalization duration and catheterization duration, overall complications, and major complications were summarized in Table 2.

**Table 2:**
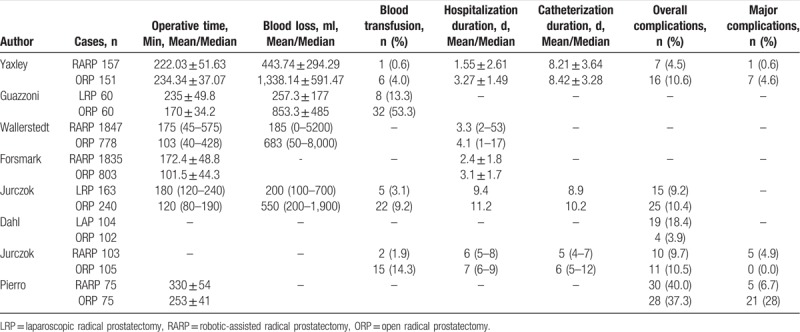
Perioperative characteristics and postoperative complications in the comparative studies between RARP/LRP and ORP.

**Figure 3:**
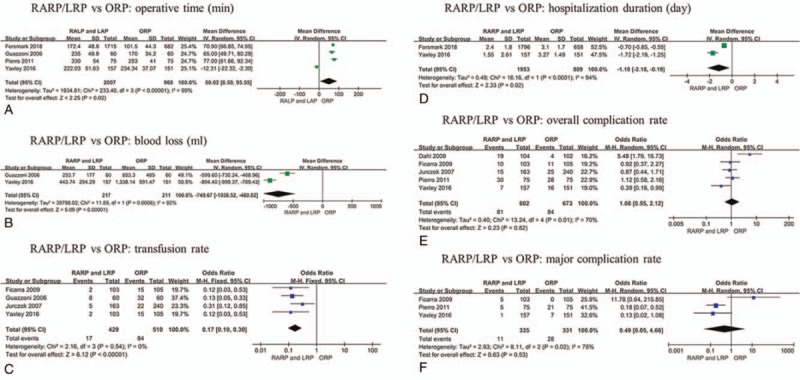
Meta-analysis outcomes of preoperative characteristics and postoperative complications comparing RARP/LRP with ORP. A. Operative time; B. Blood loss; C. Transfusion rate; D. Hospitalization duration; E. Overall complication rate; F. Major complication rate.

Four studies reported operative time in mean and standard deviations.^[27,29,34,38]^ Meta-analysis showed RARP/LRP required more operative time than ORP (MD 50.02, 95% CI 6.50 to 93.55, *P* = .02) (Fig. 3A). However, RARP/LRP resulted in lower transfusion rate (OR 0.17, 95% CI 0.10 to 0.30, *P* < 0.001), comparing to ORP, according to 4 studies^[29,35,37,38]^ (Fig. 3C). Similarly, estimated blood loss (MD −749.67, 95% CI −1038.52 to −460.82, *P* = .001) was less in RARP/LRP according to 2 studies^[29,38]^ (Fig. 3B).

Meta-analysis showed RARP/LRP required less hospitalization duration (MD −1.18, 95% CI −2.18 to −0.19, *P* = .02) based on 2 studies,^[27,29]^ comparing to ORP (Fig. 3D). Only 1 study^[29]^ reported catheterization duration in mean and standard deviations, and it showed no significant difference between 2 approaches (MD −0.21, 95% CI −1.03 to −0.61). Moreover, Jurczok et al^[37]^ and Ficarra et al^[35]^ reported the duration of catheterization was longer in ORP group.

Five studies reported overall complication rates,^[29,34–37]^ and it was 13.5% (81 out of 602 cases) for RARP/LRP and 12.5% (84 out of 673 cases) ORP, respectively. No significant difference was observed between RARP/LRP and ORP in overall complication rate (OR 1.08, 95% CI 0.55 to 2.12, *P* = .82) of pooled analysis (Fig. 3E). Similarly, there was no significant difference in major complication (Clavien grade III-V) rate (OR 0.48, 95% CI 0.05 to 4.66, *P* = .53) between the surgical techniques. The major complication rate was 3.3% (11 out of 335 cases) and 8.5% (28 out of 331 cases) for RARP/LRP and ORP, respectively (Fig. 3F).

### 3.3 Functional outcomes

It was reported before that functional outcomes between RARP and LAP are different.^[11]^ Thus, RARP and LRP studies were not merged for functional outcomes analysis. Since there are insufficient studies to compare LRP with ORP, we only compared functional outcomes between RARP and ORP.

#### 3.3.1 Erectile function

**Figure 4:**
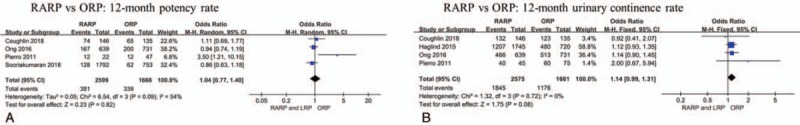
Meta-analysis outcomes of functional outcomes comparing RARP with ORP. Potency rate (A) and urinary continence rate (B) at 12 months.

The results of potency recovery at 12 months after RARP and ORP were summarized in Table 3. The definition of potency was variable among included studies. One study (3 articles) defined potency according to International Index of Erectile Function (IIEF-5),^[28]^ and it was defined as enough stiff for sexual intercourse or no/very small sexual bother (EPIC) in 3 studies,^[26,30,34]^ it was unclear in one study.^[35]^ Bilateral or unilateral nerve-sparing procedure was performed in 4 studies,^[26,28,30,34,35]^ and it was unclear in one study.^[30]^ The study which did not clearly mention the definition of potency was excluded. The potency recovery rates 12 months after RARP and ORP were 14.6% (381 of 2599 cases) and 20.3% (339 of 1666 cases), respectively. The meta-analysis showed no significant difference in potency recovery between RARP and ORP at 12 months postoperatively (OR 1.04, 95% CI 0.77 to 1.40, *P* = .82) (Fig. 4A).

**Table 3:**
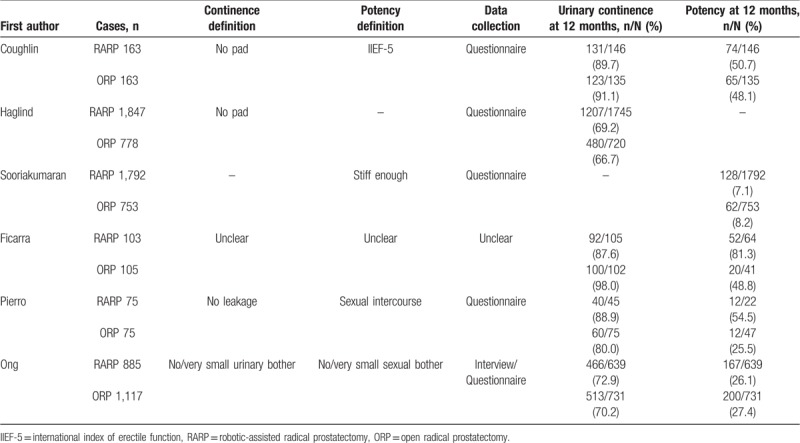
Urinary continence and potency recovery in the comparative studies between RARP and ORP.

#### 3.3.2 Urinary continence

The results of urinary continence at 12 months after RARP and ORP were summarized in Table 3. The urinary continence was reported in 5 studies.^[28,30,32,34,35]^ Of these studies, 4 studies defined urinary continence with no pad use or no leakage, another study^[30]^ defined it as patients with no/very small urinary bother. One study in which the definition is unclear was excluded.^[35]^ The urinary continence rates 12 months after RARP and ORP were 71.6% (1845 of 2575 cases) and 70.8% (1176 of 1661 cases), respectively. No significant difference was observed in urinary continence between RARP and ORP at 12 months postoperatively (OR 1.14, 95% CI 0.99 to 1.31, *P* = .08) (Fig. 4B).

### 3.4 Oncological outcomes

#### 3.4.1 Positive surgical margin

**Figure 5:**
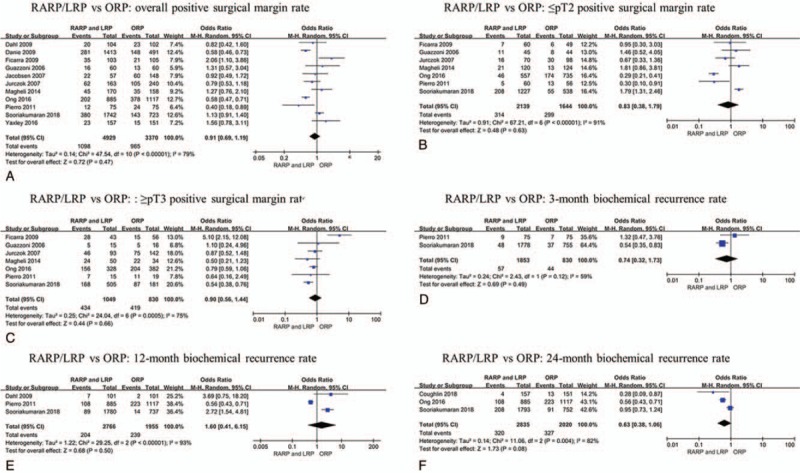
Meta-analysis outcomes of oncological outcomes comparing RARP/LRP with ORP. A. Overall PSM rate; B. ≤pT2 PSM rate; C. ≥pT3 PSM rate; Overall BCR rate at 3 (D), 12 (E), 24 (F) months.

The PSM rate for RARP/LRP and ORP were summarized in Table 4. PSM results were reported in all studies, and seven of them reported pathological stage of PSM results. Overall PSM rate of RARP/LRP was 22.3% (1098 of 4929 cases), which is similar with that of ORP (28.6%, 965 of 3370 cases). There is no significant difference in overall PSM rate (OR 0.91, 95% CI 0.69 to 1.19, *P* = .47) between RARP/LRP and ORP (Fig. 5A). When PSM was analyzed in subgroup, PSM rate of RARP/LRP and ORP were 14.7% (314 of 2139 cases) and 18.8% (299 of 1644 cases) in ≤pT2 tumors, respectively, with a pooled OR of 0.83 (95% CI 0.38 to 1.79, *P* = .63) (Fig. 5B). While in ≥pT3 tumors, the PSM rate were 41.4% (434 of 1049 cases) and 50.1% (419 of 830 cases) for RARP/LRP and ORP, respectively, with a pooled OR 0.90 (95% CI 0.56 to 1.44, *P* = .66) (Fig. 5C).

**Table 4:**
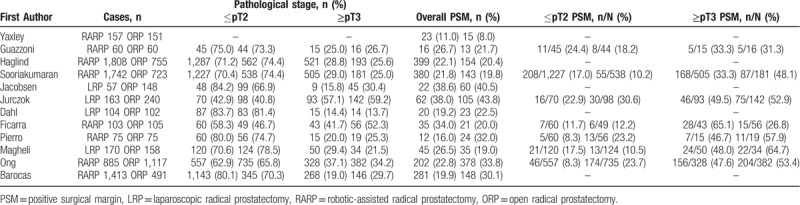
Positive surgical margin rates in the comparative studies between RARP/LRP and ORP.

### 3.5 Biochemical recurrence

The BCR rate for RARP/LRP and ORP were summarized in Table 5. BCR results were reported in 5 studies,^[26,28,30,34,36]^ all these studies defined PSA ≥0.2 ng/ml as BCR, with a follow-up of 3, 12, and 24 months. The BCR rates were 3.1% (57 of 1853 cases), 7.4% (204 of 2766 cases) and 11.3% (320 of 2835 cases) at 3, 12, and 24 months after RARP/LRP, respectively. Similarly, the BCR rates were 5.3% (44 of 830 cases), 12.2% (239 of 1955 cases) and 16.2% (327 of 2020 cases) at 3, 12, and 24 months after ORP, respectively. There were no significant differences in BCR rates between RARP/LRP and ORP at 3 (OR 0.74, 95% CI 0.32 to 1.73, *P* = .49), 12 (OR 1.60, 95% CI 0.41 to 6.15, *P* = .50), 24 (OR 0.63, 95% CI 0.38 to 1.06, *P* = .08) months postoperatively (Fig. 5D–F).

**Table 5:**
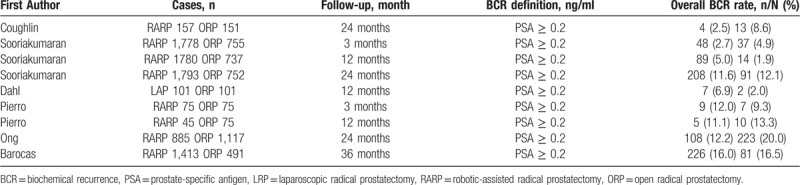
Biochemical recurrence rates in comparative studies between RARP/LRP and ORP.

### 3.6 Patient cost

Only Forsmark et al^[27]^ reported patient cost comparing RARP and ORP. The mean per patient cost were $15,974 (95% CI, 15,405 to 16,543, *P* < .001) and $12,137 (95% CI, 11,122 to 13,152, *P* < .001) in RARP and ORP groups, respectively. The cost mean difference between RARP and ORP was $ 3837 (95% CI, 2747 to 4928, *P* < .001).

#### 3.6.1 Publication bias

Visual inspection of the funnel plot for operative time, estimated blood loss, hospitalization duration, overall complication rate, major complication rate, urinary continence, urinary potency, overall BCR, overall PSM, ≤pT2 PSM rate, ≥pT2 PSM rate revealed asymmetry (data not shown), but publication bias cannot be excluded due to insufficient studies.

## 4 Discussion

With the rapid development of laparoscopic techniques, RARP and LRP have become an alternative procedure rather than ORP, and RARP and LRP have been used widespread in many countries^[41–43]^. Sood et al^[44]^ and Descazeaud et al^[45]^ even believed RARP is inching toward gold standard for radical prostatectomy. In contrast to the wide application of RARP and LRP worldwide, scarce definitive evidences are insufficient to conclude the superiority of RARP/LRP over ORP. Subsequently, both EAU^[4]^ and AUA^[3]^ guidelines do not recommend RARP and LRP over ORP. Several systematic reviews including retrospective studies have been completed,^[11,12,14–19]^ which showed consistent results that RARP/LRP yielded less blood loss and blood transfusion rates, hospitalization duration. However, they were inconsistent in functional and oncological outcomes. The quality of evidences based on retrospective studies is relatively low.^[22,46]^ To date, only one systematic review including only RCTs comparing RARP/LRP and ORP are available,^[10]^ but it is still limited due to small samples. By including all prospective comparative studies, our comprehensive systematic review demonstrated that RARP/LRP were safe approaches, with similar functional and oncological outcomes as ORP, moreover, they were associated with lower blood loss and transfusion rate, hospitalization duration.

The perioperative outcomes comparing RARP/LRP and ORP including estimated blood loss, operative time, transfusion rate, hospitalization duration, catheterization duration, overall complications and major complications. According to the data collected from prospective comparative studies, ORP required less operative time, which is consistent with the meta-analysis conducted by Carlo et al^[12]^ in 2014. Our findings demonstrated that RARP/LRP were related to lower transfusion rate and less blood loss, comparing to ORP, which is consistent with all systematic reviews conducted previously.^[10–12,15,19]^ Hospitalization duration from only 2 studies were included in this studies, and the result indicated that RARP and LRP shorten hospitalization duration, comparing to ORP. This is also supported by several other studies^[27,35,36]^ which were not included in the pooled analysis. Ficarra et al^[19]^ and Carlo et al^[12]^ also showed similar results. Yaxley et al^[29]^ reported that RARP and ORP showed similar catheterization duration; however, Jurczok et al^[37]^ and Ficarra et al^[35]^ suggested catheterization duration was in favor of RARP/LRP, rather than ORP. These conflicted results support more high-quality studies to conclude it. No significant differences between 2 RARP/LRP and ORP were observed. Nevertheless, Schmitges et al^[47]^ and Jaffe et al^[48]^ demonstrated complication rate decreased after RARP/LRP over time, and this has also been observed in bladder cystectomy.^[49]^ This implies that complications are more concerned with individual surgeons, but not surgical approaches.

The potency and urinary continence are the most important functional outcomes after radical prostatectomy. Our meta-analysis indicated potency recovery rate and urinary continence rate at 12 months after RARP and ORP are similar. The result suggested that RARP can provide comparable functional outcomes, similar as ORP. Nevertheless, the results should be interpreted cautiously. Firstly, the results were pooled based on small sample sizes from 4 studies. Furthermore, the definition of potency and urinary continence were variable. Finally, surgical skills such as nerve-sparing technique, bladder neck preservation and posterior musculofascial reconstruction were associated with urinary potency and continence outcomes.^[17,50,51]^ It is impossible to rule out these influence factors and analyze in subgroup in the present meta-analysis.

PSM is a very important oncological outcome for localized prostate cancer, and it is associated with BCR and initiation of adjuvant treatment. The overall PSM rates between RARP/LRP and ORP in this meta-analysis are comparable. When PSM was analyzed in subgroup, no significant differences were observed in ≤pT2 and ≥pT3 tumors between 2 approaches. These were consistent with previous systematic reviews.^[11,16]^ Tumor characteristics such as stage Gleason score and prostate volume, are the most relevant predictors with PSM,^[16,52]^ and it is in line with the present meta-analysis. ≥pT3 tumors contributed more PSMs, comparing to ≤pT2 tumors, in both surgical approaches. BCR is an important oncological parameter to reveal the safety of surgical approach. Few systematic reviews reported BCR before, as the lack of relevant studies. Novara et al^[16]^ compared BCR in RARP and ORP, and no significant differences in BCR-free survival was demonstrated between RRP and RARP. The BCR rates between RARP/LRP and ORP are all comparable at different follow-ups. Barocas et al^[39]^ and Drouin et al^[53]^ reported 3-year BCR-free survival rate and 5-year BCR-free survival rate were also similar between RARP and ORP groups, respectively. Therefore, RARP/LRP are safe approaches based on BCR results. Nevertheless, we should treat these results with some caution. Part of the patients in both groups received adjuvant therapies after radical prostatectomy, and it decreased the BCR rates. It is unclear whether adjuvant therapies contributed similarly between 2 approaches of BCR rate. Further studies with longer follow-up, which can rule out the influence of adjuvant therapies, are required.

Few studies compared direct costs of different approaches to radical prostatectomy. Patient cost was higher in RARP, comparing to ORP, according to the LAPPRO study in 2018,^[27]^ which is consistent with a previous systematic review.^[13]^ RARP had the highest direct costs which may be due to increased surgical instrumentation costs. The cost is also a factor to be considered when choosing surgical approaches. Longer follow-up of patients is required to better evaluate the impact of RARP/LRP on overall costs.

Although the present systematic review concluded from the best evidence available in the literature, we still need take some potential drawbacks into consideration. It was almost impossible to assess the impact of surgeon ability on the reported results. Some studies demonstrated that heterogeneity between surgeons resulted in oncologic and functional variability.^[54,55]^ Our comparative analyses were not adjusted for the baseline differences in patient characteristics and surgical experience. It is likely that the characteristics of the patients included in the comparative studies, as well as the experience of the surgeons, were not always comparable in the different arms. This present systematic review included only 2 RCTs (3 articles),^[28,29,38]^ which would decrease the evidence level. Moreover, most of the included studies did not adopt accurate methodology for reporting complications. Strong evidence of heterogeneity and insufficient studies could also result in some bias in our study.

## 5 Conclusion

This systematic review demonstrates RARP/LRP are followed by significantly lower blood loss and transfusion rate, shorter hospitalization duration, supporting that RARP/LRP are safe surgical approaches, comparing to ORP, but it does not show the superiority of any surgical approach in terms of post-operative complications, functional and oncologic outcomes. Due to the lack of available RCTs, further high-quality, multicenter RCTs with long-term follow-up are required for more evidence.

## Author contributions

**Conceptualization:** Minfeng Chen.

**Data curation:** Lan Cao, Zhenyu Yang.

**Formal analysis:** Lan Cao, Zhenyu Yang.

**Investigation:** Lan Cao, Zhenyu Yang.

**Methodology:** Lin Qi, Minfeng Chen.

**Project administration:** Minfeng Chen.

**Software:** Lan Cao, Zhenyu Yang.

**Supervision:** Lin Qi, Minfeng Chen.

**Writing – original draft:** Lan Cao, Zhenyu Yang.

**Writing – review & editing:** Lin Qi, Minfeng Chen.

Minfeng Chen orcid: 0000-0002-9759-9894.
